# Major influence of CD4 count at the initiation of cART on viral and immunological reservoir constitution in HIV-1 infected patients

**DOI:** 10.1186/s12977-016-0278-5

**Published:** 2016-06-30

**Authors:** Anne-Emmanuelle Depincé-Berger, Delphine Vergnon-Miszczycha, Alexandre Girard, Anne Frésard, Elisabeth Botelho-Nevers, Claude Lambert, Emilie Del Tedesco, Christian Genin, Bruno Pozzetto, Frédéric Lucht, Xavier Roblin, Thomas Bourlet, Stéphane Paul

**Affiliations:** Groupe Immunité des Muqueuses et Agents Pathogènes – GIMAP EA 3064, CIC 1408, Faculté de Médecine J. Lisfranc, Université de Saint-Etienne, Université de Lyon, 42023 Saint-Étienne Cedex 02, France; Laboratoire d’Immunologie, Centre Hospitalo-Universitaire, Saint-Étienne, France; Service de Maladies Infectieuses et Tropicales, Centre Hospitalo-Universitaire, Saint-Étienne, France; Service d’Hépato-Gastroentérologie, Centre Hospitalo-Universitaire, Saint-Étienne, France; Service des Agents Infectieux et d’Hygiène, Centre Hospitalo-Universitaire, Saint-Étienne, France

**Keywords:** HIV, cART, GALT, Homing, Reservoir

## Abstract

**Background:**

A persistent immune activation is observed in gut during HIV-1 infection, which is not completely reversed by a combined antiretroviral therapy (cART). The impact of the time of cART initiation may highly influence the size of the viral reservoir and the *ratio* of CD4^+^/CD8^+^ T cells in the gut. In this study, we analyzed the characteristics of HIV rectal reservoir of long-term treated patients, regarding their blood CD4^+^ T cells count at the time of cART initiation.

**Results:**

Twenty-four consenting men were enrolled: 9 exhibiting a CD4^+^ T cells count >350/mm^3^ (“high-level CD4 group”) and 15 < 350/mm^3^ (“low-level CD4 group”) in blood, at the start of cART. An immunophenotypical analysis of T and B cells subpopulations was performed in blood and rectal biopsies. HIV cell-associated DNA loads and qualitative intra-cellular RNA were determined in both compartments. The *ratio* of CD4^+^/CD8^+^ T cells was significantly decreased in the blood but not in the rectum of the “low-level CD4 group” of patients. The alteration in β7^+^ CD4^+^ T cells homing was higher in this group and was correlated to a low *ratio* of CD4^+^/CD8^+^ T cells in blood. An initiation of cART in men exhibiting a low-level CD4 count was also associated with an alteration of B cells maturation. HIV blood and gut DNA reservoirs were significantly lower in the “high-level CD4 group” of men. A high HIV DNA level was associated to a detectable intracellular HIV RNA in rectum.

**Conclusions:**

An early initiation of cART could significantly preserve gut immunity and limit the viral reservoir constitution.

**Electronic supplementary material:**

The online version of this article (doi:10.1186/s12977-016-0278-5) contains supplementary material, which is available to authorized users.

## Background

Although combined antiretroviral therapy (cART) has dramatically improved the quality of life and lifespan of HIV infected individuals, it still fails to eliminate viral reservoirs. The Gut Associated Lymphoid Tissue (GALT) is the largest reservoir of HIV-1, as it harbors most of HIV target cells as activated memory CD4^+^/CCR5^+^ T cells [1, 2]. Intestinal T and B cells express α4β7 integrin, a gut mucosal homing receptor which binds to gp120 HIV-1 envelope, facilitating the infection of intestinal T cells and the early establishment of the gut HIV reservoir [3]. Intensive viral replication in the GALT leads to an early impairment of mucosal immunity, due to the severe CD4^+^ T cells depletion, that could also be explained by a lack of recruitment in the gut [4, 5]. Among T cells, IL-17 secreting CD4^+^ T cells (Th17) are particularly depleted during HIV infection [6]. This depletion could be associated with HIV progression since these cells play a crucial role in the maintenance of mucosal immunity [6]. This proinflammatory activity is balanced by regulatory T cells (Treg) and a dysbalance of the Th17/Treg *ratio* may reflect the loss of the intestinal epithelial barrier integrity [7, 8]. During HIV infection, Treg cells are highly recruited into the GALT, which increase the imbalance with Th17 cells [9]. These damages are responsible for an increase in microbial translocation, which is associated with immune activation and progression to AIDS [10–14]. B cells compartment has been poorly studied but it seems to be affected very early during HIV infection, with a global hyperactivity, partially reversed by cART [15, 16]. However, the influence of cART on B cells rescue remains unknown [15].

Several recent studies have shown that cART initiation during acute or early HIV-1 infection reduces HIV DNA reservoir size and improves the *ratio* of CD4^+^/CD8^+^ T cells in blood [17–20]. Post-treatment controllers (PTc), who started long-term cART early after HIV infection, have very low levels of HIV DNA in peripheral blood mononuclear cells (PBMC), similarly to elite controllers [21]. Unlike most HIV-infected individuals, they maintain an undetectable plasmatic viral load after several years of cART interruption, suggesting that a weak reservoir is a prerequisite to achieve a functional cure [21]. By extrapolation, it could be hypothesized that the gut viral reservoir is also decreased and that mucosal immunity is restored when cART is initiated during the primary phase of infection. In a monkey lab model, the initiation of suppressive cART 3 days after the infection blocked the emergence of viral RNA and proviral DNA in blood and reduced the size of viral reservoir in lymph nodes and GALT [22].

The gut viral reservoir begins to form within the first days after HIV exposure, and grows during acute HIV infection [23]. Similarly, intestinal T cells are depleted directly after infection [5], due to high viral replication, host immune response and bystander effects [24]. Most studies also concluded that long-term and optimal treatment can’t fully restore mucosal immunity [25]. These observations led us to study the impact of time of cART start on the size of viral reservoir and on the *ratio* of CD4^+^/CD8^+^ T cells in the gut. For this, we analyzed the virological and immunological characteristics of the rectal HIV reservoir of long-term treated patients, regarding their blood CD4^+^ T cells count at the time of cART initiation.

## Results

Twenty-four men were enrolled between May 2013 and March 2015. For 9 patients (“high-level CD4 group”), cART was initiated when the number of CD4^+^ T cells was over 350 CD4^+^ T cells/mm^3^ [398–1025/mm^3^]. For 15 (“low-level CD4 group”), cART was initiated when the number of CD4^+^ T cells was below 350 CD4^+^ T cells/mm^3^ [14–347/mm^3^]. The number of 350 CD4^+^ T cells/mm^3^ was considered, since it has been suggested that mortality and disease progression is reduced if cART is started in patients exhibiting blood CD4^+^ T cells load above this threshold [26]. The cART was initiated during primary HIV infection (PHI, Fiebig III score [27]) in four patients, three belonging to “high-level CD4 group”, and one to “low-level CD4 group”. Detailed results of their immunophenotypical and virological markers are shown in Table 1.

**Table 1:** Main results of immunophenotypical and virological reservoir analysis of patients enrolled in the study according to their CD4^+^ cells count at the time of cART initiation

	Blood CD4^+^ T count at the time of diagnosis (cells/mm^3^)		p value
	>350 “high-level CD4 group” (n = 9)	<350 “low-level CD4 group” (n = 15)	
Immunophenotypical analysis of T cells			
MFI CD3^+^/PD1^+^ T cells			
Blood	17.8 [16.9–28]	17.7 [16.5–22]	0.79
Rectum	30.9 [23.9–52.2]	38.8 [27.5–44.6]	0.88
p24^+^/CD4^+^ T cells (% of CD4^+^ T cells)			
Blood	0.71 [0.26–1.45]	0.92 [0.39–1.59]	0.55
Rectum	8.62 [2.97–16.72]	7.23 [2.42–27.46]	0.96
MFI CXCR4 in CD4^+^ T cells			
Blood	17.78 [15.03–24]	17.33 [16.69–21.74]	0.72
Rectum	17.44 [15.42–19.13]	16.15 [16.02–18.61]	0.46
MFI CCR5 in CD4^+^ T cells			
Blood	42.03 [37.82–46.63]	40.75 [38.47–46.23]	0.97
Rectum	51.01 [43.41–65.4]	52.64 [45.02–74.96]	1
Th17 cells/Treg *ratio*			
Blood	2.9 [1.4–4.3]	2.2 [1.5–3]	0.64
Rectum	1.4 [1.1–2.8]	1.3 [1.1–2.5]	0.96
Blood/rectum *ratio* of β7^+^ expression in CD4^+^ T cells	0.9 [0.8–1.2]	1.6 [1.3–1.8]	0.0006
Blood/rectum *ratio* of β7^+^ expression in CD8^+^ T cells	0.7 [0.5–1.2]	0.8 [0.7–1]	0.54
MFI CCR9 in CD4^+^ T cells			
Blood	9.67 [9.29–10.65]	10.13 [9.27–11.25]	0.6
Rectum	18.19 [13.93–23.51]	20.02 [16.05–25.23]	0.6
MFI CCR9 in CD8^+^ T cells			
Blood	14.03 [13.01–20.02]	13.73 [13.19–17.29]	0.82
Rectum	19.11 [16.41–24.14]	16.36 [14.78–18.78]	0.12
Naive CD4^+^ T cells (% of CD4^+^ T cells)			
Blood	33.48 [27.55–46]	28.63 [17.03–42.8]	0.36
Rectum	5.07 [1.96–10.35]	4.99 [3.54–7.12]	0.73
CM CD4^+^ T cells (% of CD4^+^ T cells)			
Blood	38.29 [31.37–44.25]	37.06 [26.74–48.41]	0.97
Rectum	24.38 [21.39–41.73]	15.42 [12.75–41.48]	0.39
EM CD4^+^ T cells (% of CD4^+^ T cells)			
Blood	23.83 [11.89–27.31]	22.59 [12.78–33.74]	0.55
Rectum	66.27 [53.45–71.44]	67.99 [50.44–82.32]	0.49
EMRA CD4^+^ T cells (% of CD4^+^ T cells)			
Blood	4.83 [1.73–8.08]	2.23 [0.55–4.53]	0.22
Rectum	0.61 [0.45–1.77]	0.39 [0.12–5.2]	0.66
Immunophenotypical analysis of B cells			
Blood naive IgD^+^ B cells (% of conventional B cells)	76.6 [60.6–79.1]	82.2 [77.3–87.6]	0.05
Blood activated IgD^−^ B cells (% of conventional B cells)	23.1 [20.8–38.8]	17.6 [12.4–22.4]	0.05
Memory B cells (% of conventional B cells)	5.94 [1.68–18.05]	4.05 [2.07–5.66]	0.34
Virological reservoir analysis			
HIV DNA load (log_10_ copies/millions of cells)			
Blood	2.41 [2.03–2.93]	3.48 [3.17–3.7]	0.01
Rectum	3.06 [1.93–3.63]	3.6 [3.4–4.08]	0.04

Medians of MFI or percentages [range]

*CM* central memory, *EM* effector memory, *EMRA* effector memory T cells expressing RA

### Initiation of cART with high level of CD4 is associated with an elevation of the ratio CD4^+^/CD8^+^ T cells in blood but not in GALT (Fig. 1 and Additional file 1: Figure S1 and Additional file 2: Fig. S2)

**Fig. 1:**
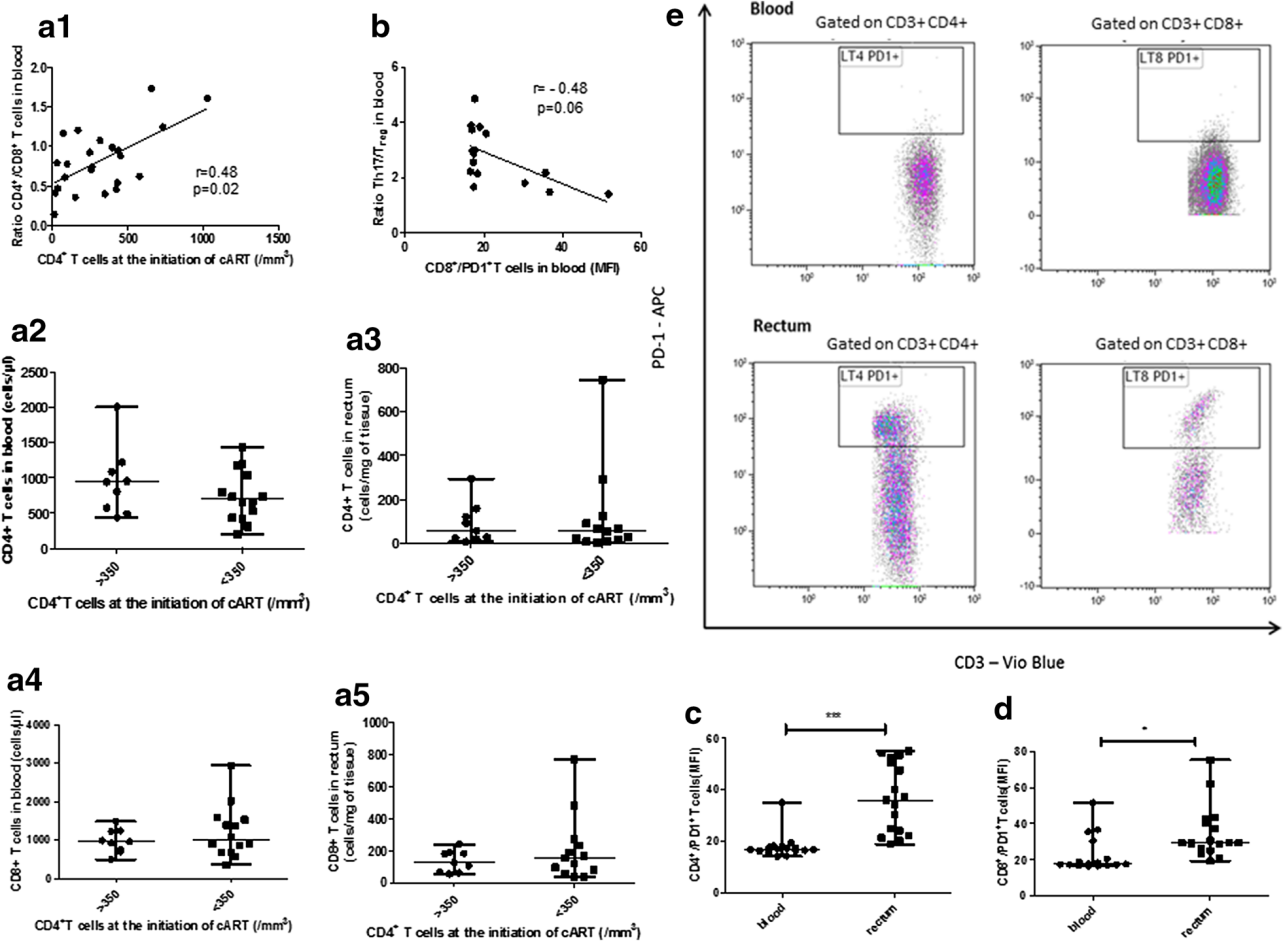
Initiation of cART with higher CD4 level preserves CD4^+^ T cells in blood and exhaustion marker PD-1 reveals a defect of the gut barrier integrity. Correlation between CD4^+^ T cells at the initiation of cART and *ratio* CD4^+^/CD8^+^ T cells in blood (**a**_**1**_) or with absolute numbers of CD4^+^ T cells in blood (**a**_**2**_) and rectum (**a**_**3**_) or CD8^+^ T cells (**a**_**4**_, **a**_**5**_). Correlation between PD-1 expression on CD8^+^ T cells (MFI) in blood and Th17/Treg *ratio* in blood (**b**). Differences of PD-1 expression (MFI) in CD4^+^ and CD8 ^+^ T cells in blood and rectum (**c**, **d**). Flow cytometry analysis of this marker in CD4^+^ and CD8^+^ T cells (**e**). *Gating strategy*: lymphoid cells were gated using the FSC/SSC parameters. Then, T cells were gated by their expression of CD3 and were separated in CD4^+^ and CD8^+^ T cells by the CD4 expression and PD1 expression was studied in CD3^+^/CD4^+^ and CD3^+^/CD8^+^ T cells. *Horizontal lines* represent median values and ranges. *p < 0.05; ***p < 0.0001. Each *symbol* represents an individual patient

Overall, the *ratio* CD4^+^/CD8^+^ T cells was significantly higher in blood than in rectum (0.8 [0.5–1.1] vs 0.4 [0.2–0.6], p = 0.0023). In blood, it was significantly correlated to the CD4^+^ T cells count at the initiation of cART (r = 0.48, p = 0.02) (Fig. 1a_1_), but not in rectum (r = 0.28, p = 0.21). The exhausted/hyperactivated phenotype of T cells (PD1^+^) was higher in the rectum than in the blood (MFI of the gated CD4^+^/PD1^+^ population 35.9 [23.1–51.4] vs 16.9 [16.2–17.8], p < 0.0001 and MFI of the gated CD8^+^/PD1^+^ population 29.4 [25.9–41.6] vs 17.7 [17.1–27.9], p = 0.002; Fig. 1c–e) and was not significantly different between the two groups (MFI of CD3^+^/PD1^+^ T cells 17.8 [16.9–28] vs 17.7 [16.5–22], p = 0.79 in blood and MFI 30.9 [23.9–52.2] vs 38.8 [27.5–44.6], p = 0.88 in rectum). The proportion of p24^+^CD4^+^ T cells (among CD4^+^ T cells) was not significantly different between the 2 groups, in both compartments (0.71 % [0.26–1.45] in “high-level CD4 group” vs 0.92 % [0.39–1.59] in “low-level CD4 group”, p = 0.55, in blood and 8.62 % [2.97–16.72] in “high-level CD4 group” vs 7.23 % [2.42–27.46] in “low-level CD4 group”, p = 0.96, in rectum). The same findings were observed for the expression of CXCR4 co-receptor (MFI CXCR4 17.78 [15.03–24] in “high-level CD4 group” vs 17.33 [16.69–21.74] in “low-level CD4 group”, p = 0.72, in blood and MFI CXCR4 17.44 [15.42–19.13] in “high-level CD4 group” vs 16.15 [16.02–18.61] in “low-level CD4 group”, p = 0.46, in rectum) and for the expression of CCR5 co-receptor (MFI CCR5 42.03 [37.82–46.63] in “high-level CD4 group” vs 40.75 [38.47–46.23] in “low-level CD4 group”, p = 0.97, in blood and MFI CCR5 51.01 [43.41–65.4] in “high-level CD4 group” vs 52.64 [45.02–74.96] in “low-level CD4 group”, p = 1, in rectum). There was no difference of CXCR4 expression in CD4^+^ T cells between the two compartments (MFI CXCR4 17.39 [16.43–20.92] in blood vs 16.44 [16.02–18.99] in rectum, p = 0.27), whereas the expression of CCR5 co-receptor in CD4^+^ T cells was significantly higher in rectum (MFI CCR5 40.85 [37.9–45.91] in blood vs 52.64 [44.4–70.3] in rectum, p = 0.0006).

### A persistent imbalance of the Th17/Treg ratio is maintained despite a higher level of CD4 when initiating cART (Fig. 1 and Additional file 1: Figure S1)

The Th17 cells/Treg *ratio* was not different between the two groups of patients in blood (2.9 [1.4–4.3] in “high-level CD4 group” and 2.2 [1.5–3] in “low-level CD4 group”, p = 0.64) and in rectum (1.4 [1.1–2.8] in “high-level CD4 group” and 1.3 [1.1–2.5] in “low-level CD4 group”, p = 0.96). It tended to be associated to a lower exhaustion stage of CD8^+^T cells in blood (r = −0.48, p = 0.06; Fig. 1b).

### Alterations in β7^+^CD4^+^ T cells homing to the gut is higher in patients who started cART with low CD4 count (Fig. 2 and Additional file 3: Figure S3 and Additional file 4: Fig. S4)

**Fig. 2:**
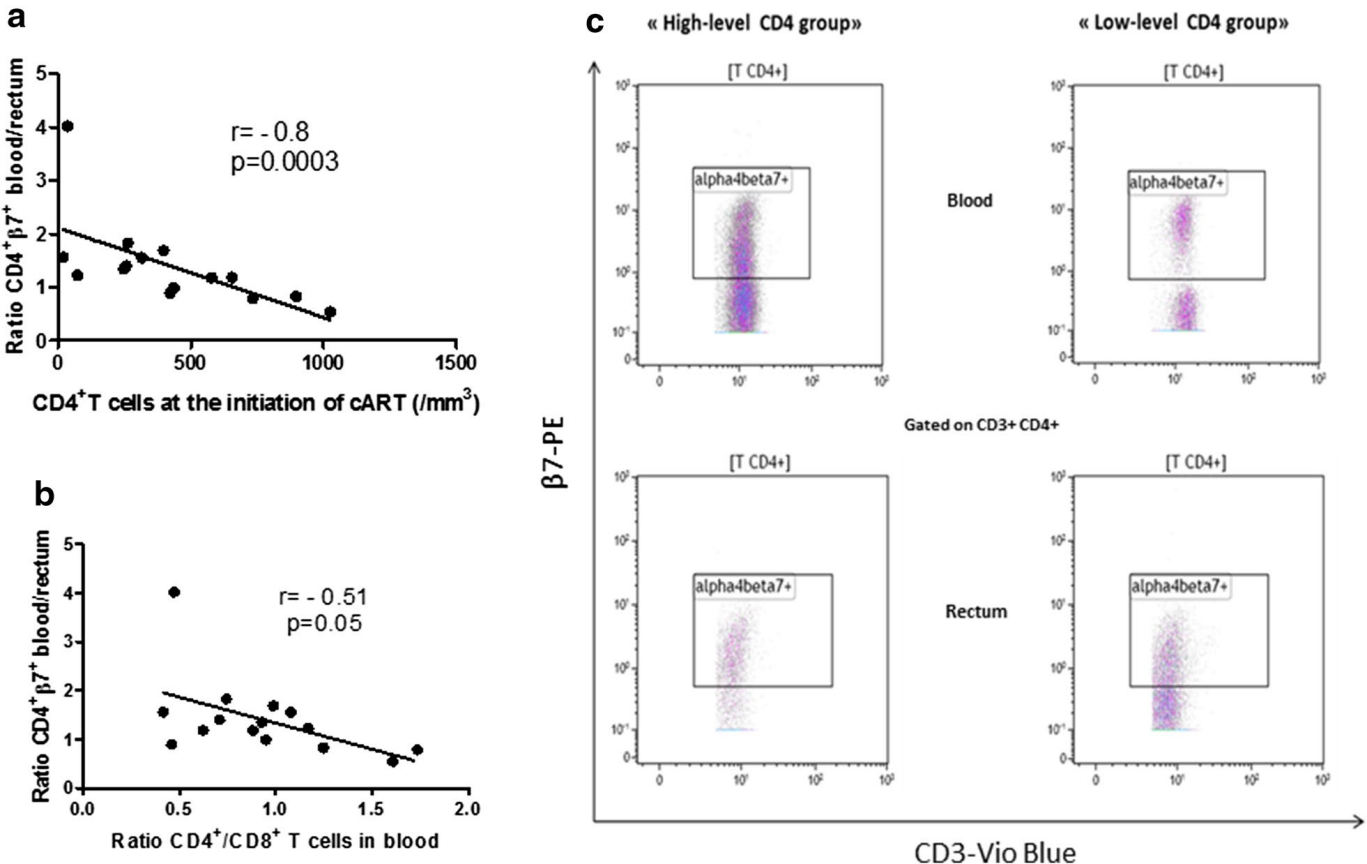
Alterations in β7^+^ CD4^+^ T cells homing to the gut is higher in patients who started cART late and is conversely correlated to the decrease of the *ratio* CD4^+^/CD8^+^ T cells. Correlation between the *ratio* blood/rectum of β7 expression in CD4^+^ T cells and the number of CD4^+^ T cells at the initiation of cART (**a**) and with the *ratio* CD4^+^/CD8^+^ T cells in blood (**b**). Flow cytometry analysis of β7 expression in CD4^+^ T cells in blood and in rectum of two HIV-infected individual representatives of “high” and “low-level CD4 group” respectively (**c**). *Gating strategy*: lymphoid cells were gated using the FSC/SSC parameters. Then, T cells were gated by their expression of CD3 and were separated in CD4^+^ and CD8^+^ T cells by the CD4 expression and β7expression was studied in CD4^+^ T cells. Each *symbol* represents an individual

The blood/rectum *ratio* of β7^+^ expression (MFI *ratio* from the gated CD4^+^/β7^+^ population) reflecting an approach to the alteration of gut homing, was calculated. It was significantly higher in patients who started the cART with low CD4 count (1.6 [1.3–1.8] vs 0.9 [0.8–1.2], p = 0.006). An inverse correlation between the CD4 level at the time of cART initiation and the alteration of β7^+^CD4^+^ T cells homing to the gut (r = −0.8, p = 0.0003) was observed (Fig. 2a). Nevertheless, there was no alteration of the gut homing of β7^+^/CD8^+^ T cells (*ratio* 0.7 in “high-level CD4 group” [0.5–1.2] vs 0.8 [0.7–1] in “low-level CD4 group”, p = 0.54). The alteration of homing to the gut could also be evaluated also by the *ratio* of β7 expression in CD3^+^ T cells in blood and total CD3^+^ T cells in rectum but there was no difference between the two groups. The blood/rectum *ratio* of β7^+^/CD4^+^ T cells was conversely associated with the *ratio* CD4^+^/CD8^+^ T cells in the blood (r = −0.51, p = 0.05) (Fig. 2b) but not in the rectum (r = 0.14, p = 0.61). CCR9 gut homing receptor was expressed equally in both compartments (gut and blood) in the two groups. Its expression in rectal CD8^+^ T cells was not significantly higher in “high-level CD4 group” (MFI 19.11 [16.41–26.14]) versus “low-level CD4 group” (MFI 16.36 [14.78–18.78]), p = 0.12. Overall, CCR9 expression in other subtypes (rectal and peripheral CD4^+^ T cells and peripheral CD8^+^ T cells) was similar in the two groups of patients (p > 0.5).

Among CD4^+^ T cells, no difference of distribution was observed in the subtypes of maturation according to the patient’s group (naive in blood, p = 0.36, and in gut, p = 0.73; CM in blood, p = 0.97 and in gut, p = 0.39; EM in blood, p = 0.55 and in gut, p = 0.49; EMRA, p = 0.22 and in gut, p = 0.66).

### CD4 low count at the initiation of cART leads to an alteration of B cells maturation (Fig. 3 and Additional file 5: Figure S5)

**Fig. 3:**
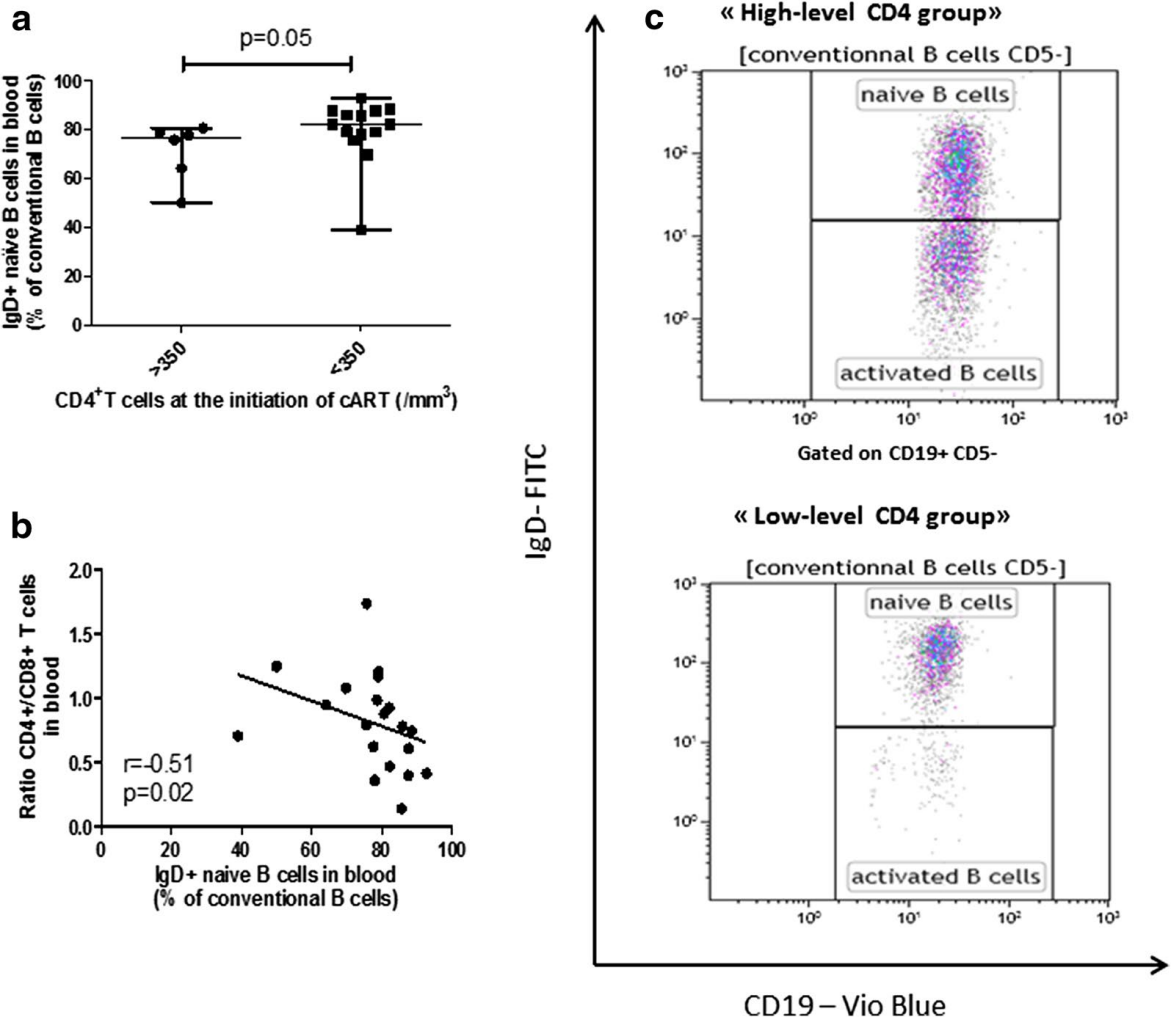
Repartition of naive and activated B cells in blood, according to the CD4^+^ T cells at the initiation of cART. Frequency of IgD^+^ naive B cells (**a**) in blood of HIV-1 infected individuals, treated above or below 350 CD4^+^/mm^3^ T cells count. *Horizontal lines* represent median values and ranges. Correlation between current CD4^+^/CD8^+^ T cells *ratio* in blood and percentage of IgD^+^ naive B cells in blood of HIV infected individuals (**b**). Each *symbol* represents an individual. Flow cytometry analysis of IgD^+^/CD19^+^/CD5^−^ B cells in the blood of two HIV-infected individual representatives of “high” and “low-level CD4 group” respectively (**c**). *Gating strategy*: lymphoid cells were gated using the FSC/SSC parameters. Then, B cells were gated by their expression of CD19 and were separated in CD5^+^ non-conventional and CD5^−^ conventional B cells. IgD expression was studied in CD5^−^ conventional B cells to plot naive and activated B cells

Proportion of non-conventional CD5^+^ B cells were not significantly different in rectum and blood (69.5 [66.2–77.8] vs 75.4 [71.5–83.9], p = 0.06). Naive IgD^+^ B cells were more represented in blood versus rectum (79.01 % of conventional B cells [75.61–85.79] vs 35.58 % [24.37–40.67], p < 0.0001) and were more frequent in blood of “low-level CD4” group patients (82.2 [77.3–87.6] vs 76.6 % [60.6–79.1], p = 0.05) (Fig. 3a) and activated B cells IgD^−^ were less represented (17.6 [12.4–22.4] vs 23.1 % [20.8–38.8], p = 0.05). Proportion of IgD^+^ naive B cells conversely correlated to the current CD4^+^/CD8^+^T cells *ratio* in blood (r = −0.51, p = 0.02; Fig. 3b). Distribution of memory B cells in blood was equivalent in the two groups (5.94 % of conventional B cells [1.68–18.05] in “high-level CD4 group” vs 4.05 % [2.07–5.66] in “low-level CD4” group, p = 0.34). Contrary to CD4^+^ T cells, no increase of β7^+^ B cells in blood of “low-level CD4” group was observed.

### Initiation of cART with high CD4 level results in a lower HIV-1 reservoir in blood and gut (Fig. 4)

**Fig. 4:**
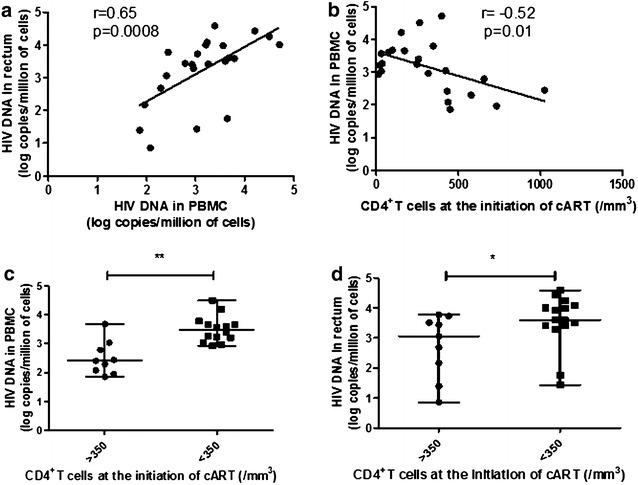
HIV reservoir size is lower in blood and rectum of patients who initiated cART with low number of CD4^+^ T cells. Correlation between HIV DNA loads in PBMC and rectum (**a**). Correlation between HIV DNA in PBMC and number of CD4^+^ T cells at the initiation of cART (**b**). Comparison between HIV DNA loads in PBMC (**c**) and rectum (**d**) of patients from the two groups. HIV DNA loads are expressed in log copies/million of cells. *Horizontal lines* represent median values and ranges.*p < 0.05; **p < 0.01. Each *symbol* represents an individual

Total HIV DNA loads in blood and rectal compartments were correlated (r = 0.65, p = 0.0008). The median HIV DNA load was not higher in rectum than in PBMCs (3.51 [2.81–3.96] vs 3.21 [2.44–3.65] log copies/millions of cells, p = 0.24). HIV DNA loads were significantly higher in “low-level” than in “high-level CD4” group (3.48 [3.17–3.7] vs 2.41 [2.03–2.93] log copies/millions of cells, p = 0.002 and 3.6 [3.4–4.08] vs 3.06 [1.93–3.63] log copies/millions of cells, p = 0.042 in blood and rectum, respectively, Fig. 4c, d). An inverse correlation between the number of blood CD4^+^ T cells at the time of cART initiation and the size of HIV reservoir in blood (r = −0.52, p = 0.01) but not in rectum (r = −0.3, p = 0.15) was observed (Fig. 4b). The patients who initiated cART during PHI presented a lower size of HIV reservoir in blood and rectum. The presence of HIV RNA was detected in 15 out of 24 (62.5 %) PBMCs and in 21 out of 24 (87.5 %) rectal biopsies, respectively. HIV-1 DNA levels were significantly higher in rectum of patients exhibiting detectable HIV-1 RNA in this compartment (3.59 [3.34–3.99] vs 1.4 [0.85–1.76], p = 0.008). This was not verified in blood compartment, but the expression of p24 in CD4^+^ T cells in PBMC tended to be higher when the virus was detected in periphery (1.03 [0.57–1.82] vs 0.39 % of CD4^+^ T cells [0.34–0.8], p = 0.09). No significant relation was observed between HIV-1 RNA detection and the *ratio* CD4^+^/CD8^+^ T cells, Th17/Treg *ratio*, β7 blood/rectum *ratio*, and PD1^+^ expression in T cells, in both compartments.

## Discussion

The CD4^+^ T cells subset depletion is higher in gut than in peripheral compartment and its recovery is delayed and incomplete under cART [5]. In our study, we showed that the *ratio* CD4^+^/CD8^+^ T cells was higher in the blood than in the rectum of cART-experienced patients and was correlated to the CD4 count at the time of initiation of therapy. A persistent activated and exhausted phenotype of PD-1 expressing T cells in GALT has been evidenced, independently of the CD4 level measured at the time of cART initiation. The role of PD-1 is defined as a negative regulator of T cell function during HIV infection, and thus as an indicator of immune activation and disease progression. It contributes to the generation of activated T cell targets for the virus itself, further driving viral replication [28, 29].

A persistent immune activation in GALT promotes early and serious immunological and structural injuries of the mucosal barrier favoring the translocation of microbial products, such as LPS, into the systemic circulation [30, 31]. Th17 cells represent a CD4^+^ T cells subset involved in enterocytes homeostasis, that plays a critical role in the mucosal defense against bacteria and fungi. Those cells are preferentially depleted in the GALT of HIV-infected patients [6]. The median counts of Th17 cells were similar in the blood and in the rectum in our two studied groups. The role of Th17 cells is balanced by Treg cells and the Th17/Treg *ratio* reflects the gut homeostasis preservation. In our study, as in others, the gut homeostasis was not significantly improved in the “high-level CD4 group”, and despite several years of cART, the restoration of CD4^+^ and Th17 cells remains typically incomplete, even in the four men who started cART during PHI [4, 32–34]. This finding is not in agreement with a previous study demonstrating a preservation of mucosal Th17 function in gut of patients who had initiated cART very early. This can be due to differences between the two studied populations, in terms of size (low in our work) and Fiebig stage at the time of cART start (FIII vs F I/II) [27]. Interestingly, in our study, gut homeostasis was negatively correlated with CD8^+^ T cell exhaustion. This could participate to increase the immune activation, probably contributing to the vicious circle “immune activation–viral replication and depletion of activated target T cells-microbial translocation”.

Another hypothesis to explain the impairment of the *ratio* CD4^+^/CD8^+^ T cells of GALT under cART is the alteration of CD4^+^ T cell homing to the gut. The α4β7 integrin is involved in gut homing of T cells via the Mucosal vascular-Addressin Cell-Adhesin Molecule 1 (MAdCAM-1) expressed on endothelial cells in gut. The ability of HIV-1 gp120 to bind to and signal through α4β7 could contribute to the tropism of HIV-1 for CD4^+^ T cells in the gut [3, 35]. As previously reported, α4β7^+^CD4^+^ T cells could be distinguished by high-level expression of integrin β7 [35]. In our study, the blood/rectum CD4^+^β7^+^ cells *ratio* reflects the alteration of gut homing CD4^+^ T cells. A later initiation of cART is significantly associated with a major gut homing alteration of CD4^+^, but not of other lymphocytes subsets (CD8^+^ T, B lymphocytes). We could hypothesize that this homing defect contributes to the CD4^+^ T cells deficiency in GALT. Nevertheless, this defect of homing was not significantly correlated with the decrease of the *ratio* CD4^+^/CD8^+^ T cells in rectum. This may be explained by the moderate number of patients in our study and by a probable peripheral α4β7 up-regulation. Mavigner et al. suggested that many of the CCR9/α4β7 expressing-CD4^+^ T cells remained in the blood circulation rather than repopulating the gut mucosa because of a lower expression of the CCL25 CCR9 ligand in the intestine of HIV-infected individuals, whereas the MAdCAM-1 expression was similar in HIV-infected patients and healthy controls [5]. We did not confirm the data of a recent paper reporting an absence of depletion of CD4^+^ cells expressing gut homing markers, in blood and in gut of cART-experienced patients [36]. However, the two works are difficult to compare since studied populations, particularly in terms of durations of infection and cART therapy, and methods (cells isolation, lymphocytes subpopulations studied) are slightly different. Interestingly, it has been suggested that the expression of α4β7 and CCR9 on activated T cells is stimulated by retinoic acid, a vitamin A metabolite produced by dendritic cells [37]. Quantifying this marker in blood could be helpful to evaluate the GALT homing ability of T cells.

As previously described, B cells hyperactivation by HIV is a common feature, characterized by hypergammaglobulinemia and a rapid turnover [16]. These abnormalities are generally reversed by cART, excepting the loss of memory B cells. However, it was suggested that an early cART initiation could partially reverse the low frequency of IgM^+^ memory B cells [38]. A similar tendency with a higher percentage of IgM^+^ memory B cells in the blood of patients treated with higher CD4 count was also noticed. Another interesting result concerns an expansion of naive B cells in the blood of our “low-level CD4 group”, to the detriment of mature B cells, very likely related to the T-cells cooperation defect in “low-level CD4 group”. A low number of CD4^+^ T cells was correlated to an expansion of naive B cells, that could lead to a production of low-specific antibodies and paucity of HIV-specific IgA response at mucosal sites [38].

One of the major obstacle to HIV eradication is the persistence of virus within infected CD4^+^ T cells in GALT and other reservoirs [39–41]. In our study, HIV DNA was detected and quantified in the rectum of all the 24 participants and for 21 of them, a persistent viral replication was detected, despite a controlled viraemia, as previously reported [42–44]. We found that patients who started cART early (during the PHI phase) exhibited a lower-size of viral reservoir in GALT and in PBMC, as previously demonstrated [17]. In PTc VISCONTI cohort, the benefit of an early treatment on the size of HIV reservoir was demonstrated [21]. Similarly, we found a significant lower HIV DNA gut reservoir in the group of men who started cART with a CD4 cells count over 350/mm^3^. In our study, a low size of viral reservoir was not significantly associated to the increase of the *ratio* CD4^+^/CD8^+^ T cells or to a loss of immune activation. The limited size of our population could explain that but Hey-Cunningham et al. reported the same findings in patients treated by HIV integrase inhibitors [45]. Without cART, «elite controllers» have abnormal levels of T cell activation despite maintaining undetectable viral loads, explained by HLA polymorphisms leading to stronger innate immune responses or lower regulatory T cell activity against HIV infection, both factors that might contribute to more-potent suppression of HIV replication [46]. Moreover, a persistent residual viraemia was reported in patients under suppressive cART, but the question whether it represents an active replication or the release of non-productive virus from the reservoir has not been resolved [47]. In our study, the hypothesis of an active replication could be suggested by the existence of a higher rectal reservoir in patients with detectable HIV RNA in gut. To better appreciate the size of the reservoir and the latent or the replicative state of the virus it would have been interesting to quantify the integrated and 2LTR forms of the virus in peripheral blood and tissues.

There are several limitations in our study, concerning first its low statistical power due to the limited number of patients included, especially those treated at PHI stage. Moreover, it would have been very informative to assess the evolution of the viral rectal reservoir in men during the course of HIV infection, before and after cART initiation. Obviously, for ethical reasons it was not possible to obtain sequential biopsies in HIV infected men. Another important point is the time between infection and HIV diagnosis that is most of the time unknown but can influence the composition of the reservoir.

And finally, our study aims to explore the rectal reservoir and it is admitted that the distribution of HIV DNA and RNA in cell subsets differs in different gut compartments. This might be kept in mind when interpreting the results but an exhaustive analysis is quite complicated, since the gastrointestinal tissues are not easily accessible.

## Conclusions

In conclusion, our study suggests that early treatment partially circumvents immune dysregulation in the rectum of HIV-infected men even if their CD4^+^ cell count was elevated at the time of initiation of therapy. These findings confirm the great interest of an initiation of cART in the early stages of HIV infection to limit the viral reservoir constitution, to preserve the gut immunity and, as a consequence, to improve patients outcomes and reduce HIV transmission at the population level.

## Methods

### Patients and samples

HIV-1 chronically infected consenting men followed at the Infectious Diseases Department of the University Hospital of Saint-Etienne were enrolled. They had been under cART for more than one but <5 years, without any viral escape during this period. They also had an indication for anal cancer screening by rectoscopy. For each patient, 2 EDTA-blood samples and 4 rectal biopsies were collected at the Gastroenterology Unit of the University Hospital of Saint-Etienne. Patients were distributed in 3 groups concerning the stage of infection at the time of diagnosis, according to the CDC Classification System for HIV Infection: A (Asymptomatic or Acute HIV Infection), B (Symptomatic conditions), C (AIDS-Indicator conditions). The patient characteristics are summarized in Table 2.

**Table 2:** Comparison of the main characteristics of HIV infected-men enrolled in the study according to their CD4^+^ cells count at the time of cART initiation

	Blood CD4^+^ T count at the time of diagnosis (cells/mm^3^)		p value
	>350 “high-level CD4 group” (n = 9)	<350 “low-level CD4 group” (n = 15)	
At the time of diagnosis			
Route of contamination (%)			
MSM or bisexual	100	86.7	0.51
Heterosexual	0	13.3	0.51
Stage CDC of HIV infection (%)			
A (PHI)	62.5	6.7	0.01
B or C	0	40	0.05
At the time of blood and biopsy sampling			
Age, median in years [range]	48 [28.5–49.5]	53 [46–61]	0.03
Time since diagnosis of HIV infection, median in months [range]	40 [27.5–105.5]	44 [26–48]	0.63
Total time on cART, median in months [range]	34 [18.5–60]	42 [24–46]	0.68
cART regimen (%)			
*3 NRTI*	100	93.3	0.009
*2 NRTI/1 NNRTI*	55.5	53.3	1
*2 NRTI/1 PI*	44.4	46.6	1
*2 NRTI/1 II*	0	13.3	0.51
At the time of cART initiation			
Time since diagnosis of HIV infection, median in months [range]	2 [0.5–73.5]	0 [0–1]	0.05
Initiation at PHI stage (%)	33.3	6.7	0.13
CD4^+^ T cells/mm^3^, median [range]	450 [427–695]	98 [31–257]	<0.0001
PVL in log_10_ copies/ml, median [range]	4.97 [4.24–6.1]	5.52 [5.17–5.94]	0.25

*cART* combined antiretroviral therapy, *CDC* centers for disease control and prevention, *II* integrase inhibitors, *PHI* primary HIV infection, *PVL* plasmatic viral load, *NNRTI* non-nucleoside reverse transcriptase inhibitors, *NRTI* nucleoside reverse transcriptase inhibitors, *PI* protease inhibitors

### Tissues and cells preparation (Additional file 6: Figure S6)

PBMC were isolated from whole blood samples by standard density gradient centrifugation (Lymphoprep, Axis-Shield, Oslo, Norway), numbered and about 1 million of cells were stored at −80 °C until use. An immunophenotypical analysis was realized on the remaining fresh blood cells. Two out of four rectal biopsies were immediately frozen at −80 °C until molecular biology testing. The remaining fresh biopsies were dedicated to the immunophenotypical analysis and were first incubated in RPMI 1640 (PAA, Pasching, Austria), supplemented with 1 % of antibiotics (penicillin 100 U/ml and streptomycin 100 µg/ml, PAA) and 10 % of fetal bovine serum (Sigma Aldrich, Saint-Louis, USA). Biopsies were then crushed immediately with a sterile scalpel and a medicon of 50 µm (BD Biosciences, San Jose, USA) and 1 ml of EDTA/Versene solution (Gibco Life technologies, Paisley, UK) was added to the cellular suspension to avoid aggregates. A mechanical disruption and a filtration of the suspension were performed with a syringe equipped with a 30-gauge blunt-end needle (Nipro Europe, Zaventem, Belgium) and a sterile filter of 30 µm (BD Biosciences). Then, rectal cells were washed with RPMI-FCS and re-suspended in that medium. The quantity of cells was determined by the automated cell counter TC10 (Biorad, Hercules, CA, USA), and the viability was estimated by a trypan blue labelling.

### Immunophenotypical analysis

For each patient, an extensive immunophenotypical analysis of blood and rectal fresh cells was performed. T cells were stained with anti-CD3/VioBlue (BW264/56), anti-CD4/Viogreen (VIT4), anti-CD8/APC-Cyanine7 (BW135/80) (Miltenyi Biotec, Bergisch Gladbach, Germany). The CD4^+^ T cells/CD8^+^ T cells *ratio* was used to explore the immune dysregulation. Among T cells, regulatory cells were identified as CD25^high^ (anti-CD25/PC7, B1.49.9, Iotest Beckman Coulter, Brea, USA) and CD127^low^ (anti-CD127/FITC, MB15-18C9, Miltenyi Biotec) [48]. The CCR6 (anti-CD196/APC, 11A9, BD Biosciences) and the CD45 RA (CD45RA/PC7, 2H4LDH11LDB9, Iotest Beckman Coulter) markers were used to characterize Th17 cells [5, 49–51]. The Th17 population was defined as CD4^+^/CD45RA^−^/CCR6^+^. The Th17/Treg *ratio* was calculated. The expression of β7 integrin and CCR9 homing intestinal markers was studied on T and/or B cells (anti-β7/PE, FIB504 and anti-CDw199/APC, 112509, BD Biosciences) [5]. Different states of T cells maturation were characterized by anti-CD45RA/PC7 and CCR7 (anti-CD197/FITC, 150503, BD Biosciences): naive (CD45-RA^+^/CCR7^+^), central memory (CM, CD45-RA^−^/CCR7^+^), effector memory (EM, CD45-RA^−^/CCR7^−^) T cells and terminal effector cells expressing RA (EMRA, CD45-RA^+^/CCR7^−^) [52, 53]. Exhausted and HIV-1 infected T cells were determined with PD-1 (anti-CD279/APC, MIH4, BD Biosciences) and intracellular HIV-p24 protein expression (anti-p24/FITC, 24-4, Santa Cruz Biotechnology), respectively [54, 55]. P24 antigen staining required a permeabilization stage (BD Cytofix/Cytoperm, BD Biosciences). HIV co-receptors CXCR4 (anti-CD184/PerCP-Cy5.5, 12G5 CXCR4, BD Biosciences) and CCR5 (anti-CD195/APC-Cy7, 2D7/CCR5, BD Biosciences) were also studied.

B cells were stained with anti-CD19/VioBlue (HD37, DakoCytomation, Denmark) and anti-CD5/PC7 (BL1a, Iotest Beckman-Coulter) to distinguish conventional and non-conventional B cells. The phenotype of maturation was evaluated with anti-IgD/FITC (IADB6, Iotest Beckman Coulter), anti-IgM/APC (G20-127, BD Biosciences), anti-CD27/APC-Cy7 (M-T271, BD Biosciences) [56, 57]. Naïve (unswitched) (CD19^+^/IgD^+^), unswitched memory (CD19^+^/IgM^+^/CD27^+^) and activated (switched) (CD19^+^/IgD^−^) B cells were compared.

### Flow cytometry analysis

Analyses were performed on a FACS Canto II (Becton–Dickinson, Franklin Lakes, NJ, USA) and data were analyzed using Kaluza software (Version 1.3, Beckman-Coulter, Brea, USA). Lymphocytes were examined using forward/side-scatter gating. T cells and B cells were identified subsequently as CD3^+^ and CD19^+^ cells, within the lymphocytes population. Each tube was run until 50,000 events were recorded or the tube was exhausted. Our gating strategy was based on fluorescence minus one technique (FMO) to determine the positivity in expression of each considered surface marker.

### Total cell-associated HIV-1 DNA quantification

Total DNA was extracted from PBMCs and biopsies using the QIAamp DNA Mini Kit (Qiagen, Courtaboeuf, France). The cell-associated HIV-1 DNA was quantified by using Generic HIV^®^ DNA cell protocol (Biocentric, Bandol, France) according to the manufacturer’s instructions. The quantification of the glyceraldehyde-3-phosphate dehydrogenase (GAPDH) gene was undertaken to estimate the amount of cells tested. The results were expressed in a log of HIV DNA copies per million of cells.

### Intra-cellular HIV-1 RNA detection

Total RNA was extracted from PBMCs and biopsies using RNA Blood Mini Kit (Qiagen) and RNeasy (Qiagen), respectively. HIV-1 RNA was detected by real-time PCR using the Generic HIV^®^ assays (Biocentric), according to manufacturer’s instructions.

### Statistical analysis

Statistical analysis was performed using Prism GraphPad version 5 Software (GraphPad Prism, San Diego, CA, USA). Medians and interquartile ranges (IQR) were used to represent the average values. The non-parametric Mann–Whitney test and Chi-square test were used to determine the significance of differences between the two groups. The correlation between the level of CD4^+^ T cells at the beginning of cART and the immunological and virological characteristics of reservoirs was analyzed using the Spearman correlation test. A p value <0.05 was considered to be statistically significant.

## Additional files

10.1186/s12977-016-0278-5 Correlation between CD4^+^ T cells at the initiation of cART and CD4^+^/CD8^+^ T cells ratio in rectum **(A)**. Intensity of PD-1 expression in blood **(B)** and rectum **(C)**,Th17/Treg ratio in blood **(D)** and rectum **(E)** of patients according to their group. Horizontal lines represent median values and ranges. Each symbol represents an individual. NS: Non-Significant.
10.1186/s12977-016-0278-5 Representation of the gating strategy of PD1^+^/CD4^+^ and PD1^+^/CD8^+^ T cells. Example in the blood of a patient from the “high-level CD4 group”.
10.1186/s12977-016-0278-5 Representation of the gating strategy of beta7^+^/CD4^+^ and beta7^+^/CD4^-^ T cells. Example in the blood of a patient from the “low-level CD4 group”.
10.1186/s12977-016-0278-5 Correlation between CD4^+^/CD8^+^ T cells *ratio* in rectum and β7CD4^+^ T cells blood/rectum *ratio* **(A)** or between CD4^+^/CD8^+^ T cells *ratio* in blood and intensity of β7 expression in CD4^+^T cells in blood **(B)**. Each symbol represents an individual.
10.1186/s12977-016-0278-5 Representation of the gating strategy of naive and activated conventional B cells. Example in the blood of a patient from the “low-level CD4 group”.
10.1186/s12977-016-0278-5 Design of the Virect study.
