# Supplementary figures and images for: Major influence of CD4 count at the initiation of cART on viral and immunological reservoir constitution in HIV-1 infected patients
Source: Retrovirology. 2016 Jun 30;13:44. doi: 10.1186/s12977-016-0278-5 (PMC4929778; doi:10.1186/s12977-016-0278-5)

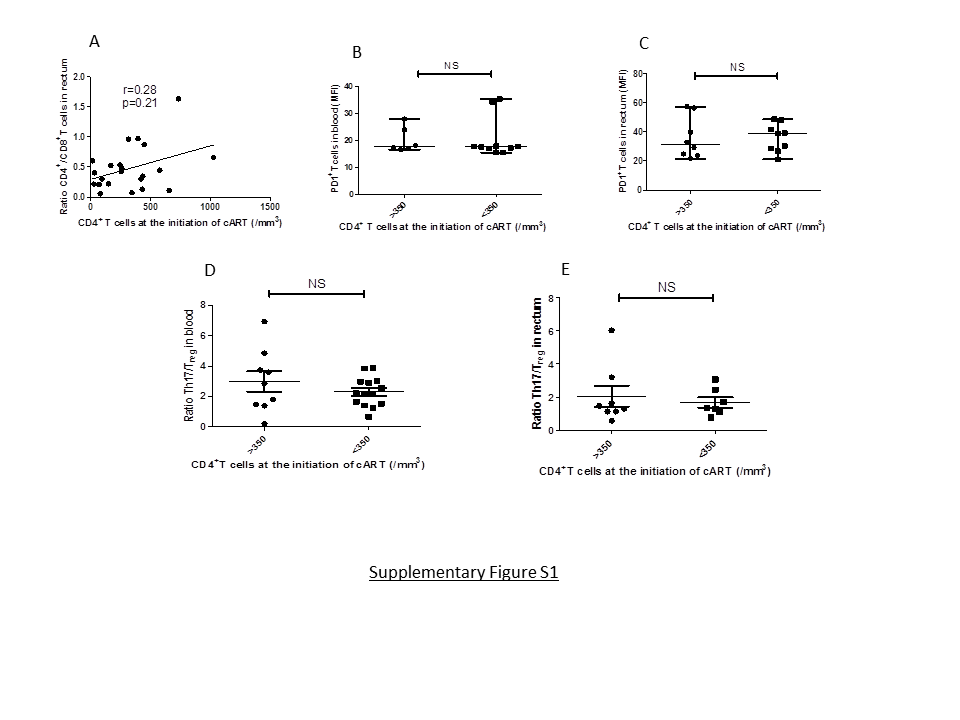

Supplement: Supplementary file 1 — 10.1186/s12977-016-0278-5 Correlation between CD4+ T cells at the initiation of cART and CD4+/CD8+ T cells ratio in rectum (A). Intensity of PD-1 expression in blood (B) and rectum (C),Th17/Treg ratio in blood (D) and rectum (E) of patients according to their group. Horizontal lines represent median values and ranges. Each symbol represents an individual. NS: Non-Significant. [file 12977_2016_278_MOESM1_ESM.tif]

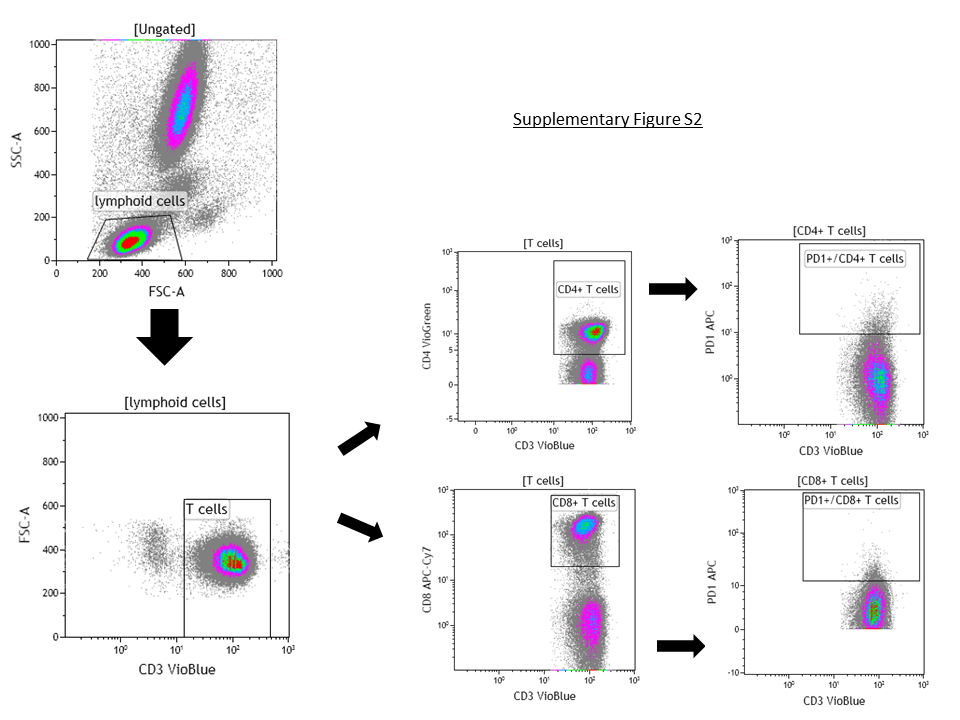

Supplement: Supplementary file 2 — 10.1186/s12977-016-0278-5 Representation of the gating strategy of PD1+/CD4+ and PD1+/CD8+ T cells. Example in the blood of a patient from the “high-level CD4 group”. [file 12977_2016_278_MOESM2_ESM.tif]

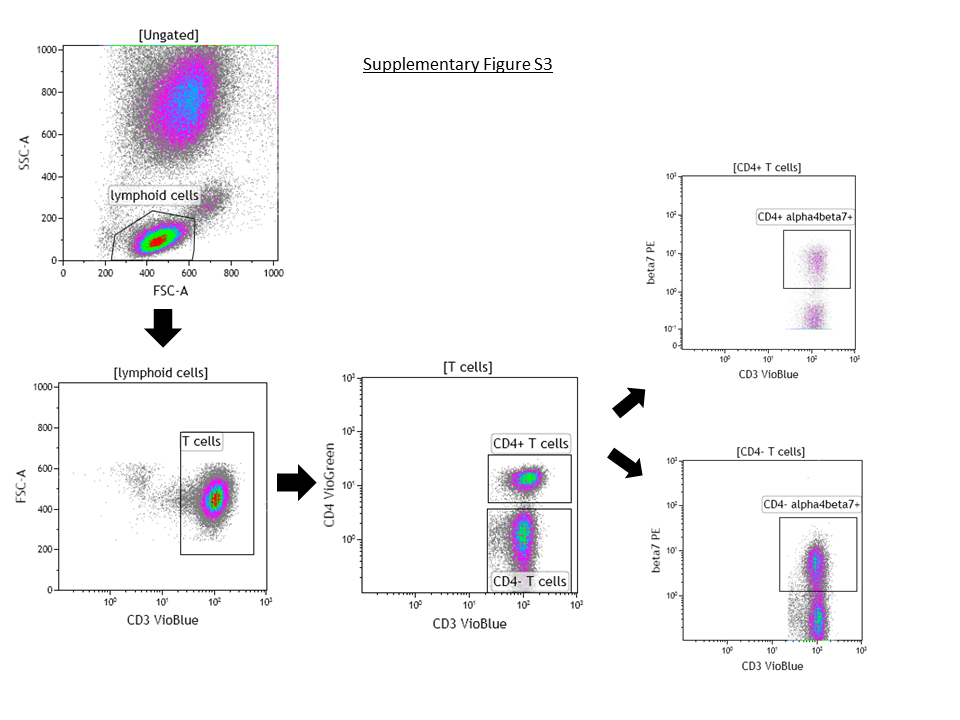

Supplement: Supplementary file 3 — 10.1186/s12977-016-0278-5 Representation of the gating strategy of beta7+/CD4+ and beta7+/CD4- T cells. Example in the blood of a patient from the “low-level CD4 group”. [file 12977_2016_278_MOESM3_ESM.tif]

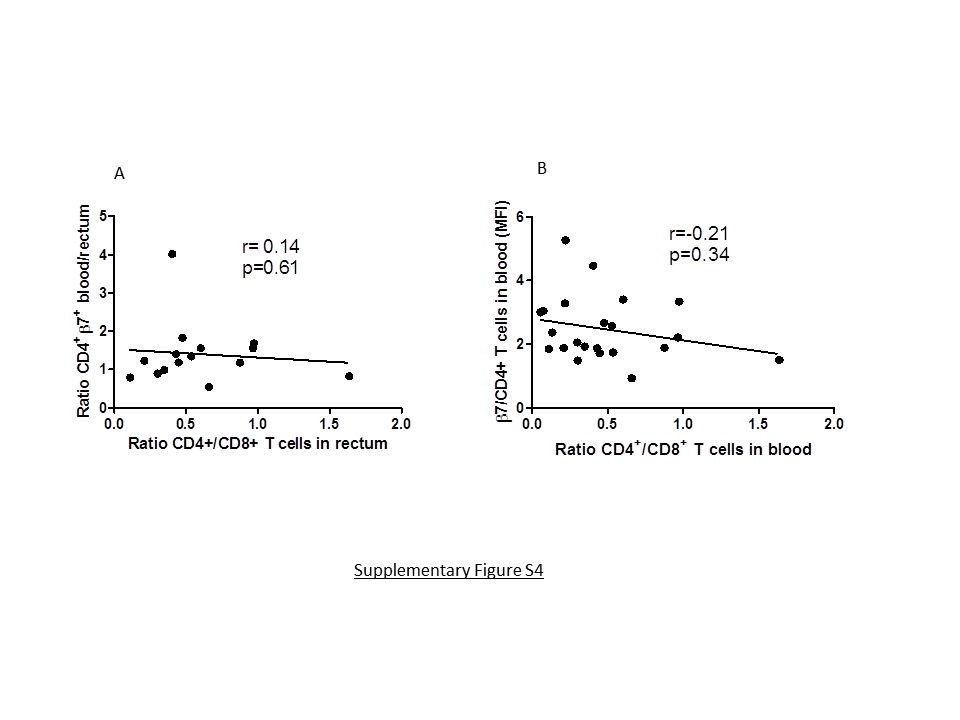

Supplement: Supplementary file 4 — 10.1186/s12977-016-0278-5 Correlation between CD4+/CD8+ T cells ratio in rectum and β7CD4+ T cells blood/rectum ratio (A) or between CD4+/CD8+ T cells ratio in blood and intensity of β7 expression in CD4+T cells in blood (B). Each symbol represents an individual. [file 12977_2016_278_MOESM4_ESM.tif]

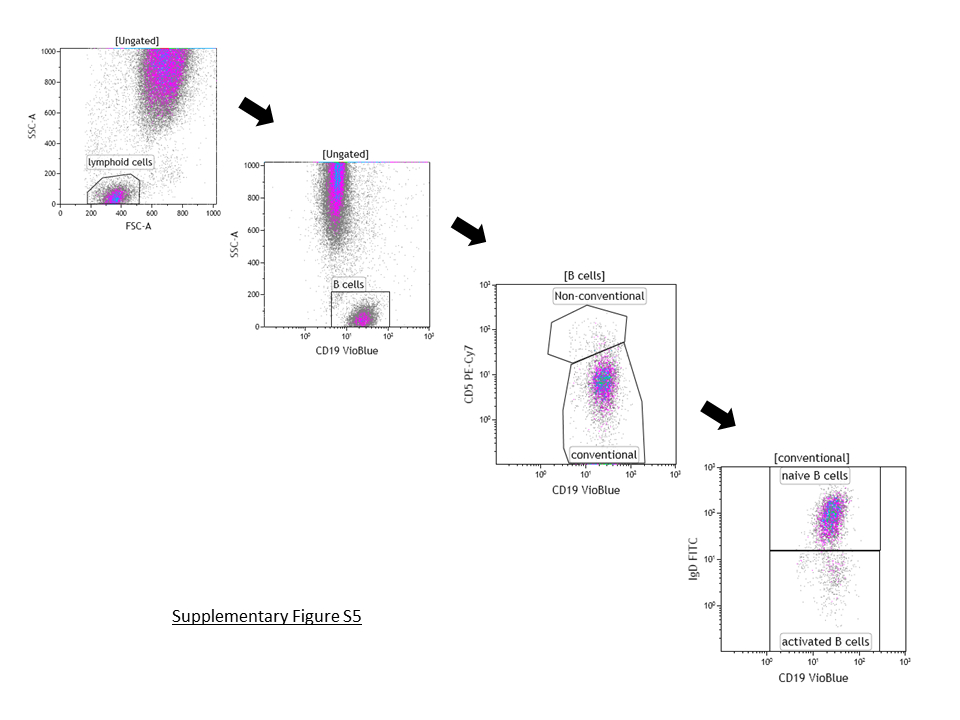

Supplement: Supplementary file 5 — 10.1186/s12977-016-0278-5 Representation of the gating strategy of naive and activated conventional B cells. Example in the blood of a patient from the “low-level CD4 group”. [file 12977_2016_278_MOESM5_ESM.tif]

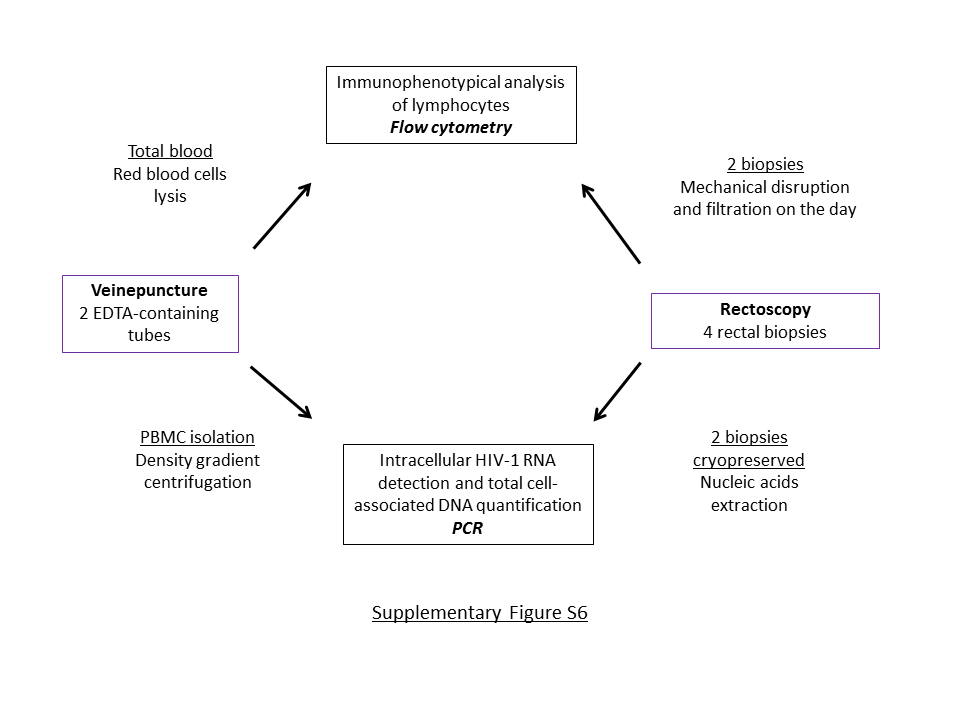

Supplement: Supplementary file 6 — 10.1186/s12977-016-0278-5 Design of the Virect study. [file 12977_2016_278_MOESM6_ESM.tif]
